# Supplementary material for: A multidisciplinary approach for investigating dietary and medicinal habits of the Medieval population of Santa Severa (7th-15th centuries, Rome, Italy)
Source: PLoS One. 2020 Jan 28;15(1):e0227433. doi: 10.1371/journal.pone.0227433 (PMC6986732; doi:10.1371/journal.pone.0227433)
Supplement: S1 Table — Each sample was associated to a code, reporting NS (for identifying the archaeological area of “Casa del Nostromo”) and a number (relative to the stratigraphic unit, SU), according to [24]. For each specimen, estimated sex (F, female; M, male; ND, sex determination was not determinable for the lack of diagnostic elements due to their bad conservation; IND, sub-adults with not determined sex because of sexual immaturity), estimated age at death (GA, generic adult individual) and district of the masticatory apparatus used for dental calculus sampling were reported. In particular, sampled teeth and relative surfaces were codified according to the Universal Teeth Numbering System [143] (B, buccal; L, lingual; M, mesial; D, distal). Finally, sample weight was indicated in grams and the analytical techniques applied on each sample were marked with an X. (DOCX) [file pone.0227433.s001.docx]

**S1 Table.** Biological proﬁle of the Medieval individuals subjected to dental calculus analyses. Each sample was associated to a code, reporting NS (for identifying the archaeological area of “Casa del Nostromo”) and a number (relative to the stratigraphic unit, SU), according to [24]. For each specimen, estimated sex (F, female; M, male; ND, sex determination was not determinable for the lack of diagnostic elements due to their bad conservation; IND, sub-adults with not determined sex because of sexual immaturity), estimated age at death (GA, generic adult individual) and district of the masticatory apparatus used for dental calculus sampling were reported. In particular, sampled teeth and relative surfaces were codiﬁed according to the Universal Teeth Numbering System [143] (B, buccal; L, lingual; M, mesial; D, distal). Finally, sample weight was indicated in grams and the analytical techniques applied on each sample were marked with an X.

| **Sample code** | **Sex** | **Age at death (Years)** | **Calculus location** | | **Sample weight (g)** | **aDNA** | **Microfossils** | **GC-MS** |
| --- | --- | --- | --- | --- | --- | --- | --- | --- |
|  |  |  | **Tooth** | **Surface** |  |  |  |  |
| **NS SU 14 Aa** | F | 19-30 | 14 | L,B | 0.023 |  | X |  |
| **NS SU 15 Ad** | ND | 31-40 | 16 | L,B | 0.025 |  | X |  |
| **NS SU 27 Ac** | M | 41-50 | 24,25  36,37,38 | L,B  B,M | 0.0125 | X | X | X |
| **NS SU 69 Ab** | M | 41-50 | 14 | B,M | 0.022 |  | X |  |
| **NS SU 78 Aa** | M | 31-40 | 38  44,45,46 | B  L,B | 0.117 | X | X | X |
| **NS SU 78 Ag** | ND | GA | 23,43 | B,M | 0.032 |  | X | X |
| **NS SU 78 Sb** | ND | GA | 13 | B,L,D | 0.019 |  | X |  |
| **NS SU 78 Sc** | IND | 13-18 | 45,46,47 | L | 0.022 |  | X |  |
| **NS SU 82** | ND | GA | 12,18,36 | B | 0.056 | X |  |  |
| **NS SU 85 Sc** | IND | 13-18 | 46,47 | B | 0.021 |  | X |  |
| **NS SU 95 Aa** | F | 19-30 | 44,45,46 | L,B | 0.103 | X | X | X |
| **NS SU 99** | F | 19-30 | 11,12,21,22 | L,B | 0.126 | X | X | X |
| **NS SU 104 Ab** | M | 31-40 | 31,32,41,42 | L | 0.095 | X | X | X |
| **NS SU 115** | F | 13-18 | 31,32,33  11,14,18  21,28 | L,B  B  B | 0.223 | X | X | X |
| **NS SU 118 A** | ND | 31-40 | 41,43,44 | L | 0.021 |  | X |  |
| **NS SU 124 Aa** | M | 31-40 | 31,32,33  36,37,38 | L,B  B | 0.099 | X | X | X |
| **NS SU 129 Aa** | M | 41-50 | 33,34,47  14,15  23,26 | B  L  L,B | 0.031 |  | X | X |
| **NS SU 129 Ac** | F | GA | 33,34,47 | L,B | 0.051 |  | X | X |
| **NS SU 130 Aa** | F | 19-30 | 46,47 | L,B | 0.023 |  | X |  |
| **NS SU 137 Ab** | M | 19-30 | 15,16,18  31,42,42 | B  L | 0.156 | X | X | X |
| **NS SU 138 Ab** | F | 41-50 | 44 | B,D | 0.025 |  | X |  |
| **NS SU 140** | F | 19-30 | 11,12,16 | B | 0.072 | X | X |  |
| **NS SU 150** | M | 19-30 | 46,47,48 | L,B | 0.104 | X | X | X |
| **NS SU 151 Aa** | M | 19-30 | 11,12,15,17  44,45 | L  B | 0.101 | X | X | X |
| **NS SU 154 Aa** | M | 31-40 | 41,42,43  46,47,48 | L,B  B,M | 0.360 | X | X | X |
| **NS SU 154 Ab** | M | 31-40 | 41,42,44  31 | L,B  L | 0.032 |  | X | X |
| **NS SU 155** | F | 31-40 | 31,32,34  36,37,21  25,26,27 | L  B  B,M | 0.295 | X | X | X |
| **NS SU 157 Aa** | F | 31-40 | 42,43,44,46,47 | L,B | 0.139 | X | X | X |
| **NS SU 158 Aa** | F | 19-30 | 33,35,36  34,37,38 | L,B  B | 0.105 | X | X | X |
| **NS SU 163 Ab** | M | 31-40 | 32,33,36,37,38 | L,B | 0.035 |  | X | X |
| **NS SU 164 Ab** | M | 19-30 | 15,26,46  33,34 | B  L,B | 0.137 | X | X | X |
| **NS SU 165** | M | 19-30 | 41,42,42  44,45,48  21,22,25 | L  L,B  L,B | 0.108 | X | X | X |
| **NS SU 168** | F | 31-40 | 16,17 | L,B | 0.071 | X | X |  |
| **NS SU 169** | F | 31-40 | 11 | L,B | 0.024 |  | X |  |
| **NS SU 177 Aa** | F | 31-40 | 26,27 | B | 0.023 |  | X |  |
| **NS SU 177 Ab** | M | 31-40 | 45,46,47  48 | B  L,B | 0.088 | X | X | X |
| **NS SU 179 Aa** | M | 41-50 | 11,14,15  16 | L,B  B | 0.092 | X | X | X |
| **NS SU 190 Aa** | M | 31-40 | 44,46,47 | L,B | 0.058 |  | X | X |
| **NS SU 190 Ad** | M | 41-50 | 35,36  37,38 | L,B  B,D | 0.170 | X | X | X |
| **NS SU 192** | M | 31-40 | 11,13 | L,B,M | 0.065 |  | X | X |
| **NS SU 193 Aa** | F | 31-40 | 31,32,41 | L,B | 0.019 |  | X |  |
| **NS SU 193 Ab** | ND | GA | 15 | B | 0.020 |  | X |  |
| **NS SU 196 Aa** | F | 41-50 | 27,28  34,37 | B  L | 0.032 |  | X | X |
| **NS SU 202** | M | 31-40 | 12,13  14,16 | L  L,B | 0.050 |  | X | X |
| **NS SU 205 Ac** | M | 19-30 | 23,33 | L,B | 0.048 |  | X | X |
| **NS SU 210** | M | 19-30 | 11,31,41 | L,B | 0.051 |  | X | X |
| **NS SU 215** | F | 13-18 | 31,32,33  36,37,38 | L  B,M | 0.082 | X | X | X |
| **NS SU 217 Aa** | M | 19-30 | 31,32,41,42  48  34,35,45  11,12,23 | L,B  M  B  L, B,M | 0.237 | X | X | X |
| **NS SU 219 Aa** | F | 31-40 | 13,14,15,16 | L,B | 0.67 |  | X | X |
| **NS SU 221 Aa** | ND | 19-30 | 16,21,22,37,45 | L,B | 0.140 | X | X | X |
| **NS SU 231** | M | 31-40 | 31,32  34,35,36 | L  L,B | 0.188 | X | X | X |
| **NS SU 235** | M | 19-30 | 11,12,13  45,46,47 | L  B | 0.361 | X | X | X |
| **NS SU 237 Aa** | M | 41-50 | 31,32,35 | L,B | 0.019 |  | X |  |
| **NS SU 240 Aa** | M | 19-30 | 31,34,35  44,45,46  21,22,26 | L,B  B,M  L,D | 0.154 | X | X | X |
| **NS SU 241** | M | 31-40 | 31,32,41,42  11,12,13  16,17,26,27 | L,B  L,B,M  B | 0.322 | X | X | X |
| **NS SU 283 Aa** | M | 19-30 | 41,42,46  21 | L  B,L | 0.114 | X | X | X |
| **NS SU 283 Ab** | ND | GA | 22 | L,B,D | 0.073 | X | X |  |
| **NS SU 284 Aa** | M | 31-40 | 41,42,43  33,36 | L  B,D | 0.040 |  | X | X |
| **NS SU 286 Aa** | M | 41-50 | 46,48 | B,M | 0.074 | X | X |  |
| **NS SU 287 Aa** | M | 31-40 | 31,32,33  34  41,42,43 | L,B  L  L,M | 0.368 | X | X | X |
| **NS SU 287 Ab** | M | 19-30 | 45,46,47,48 | L,B | 0.031 |  | X | X |
| **NS SU 287 Ac** | F | 13-18 | 45,46,47,48 | L,B | 0.089 | X | X | X |
| **NS SU 289** | M | 41-50 | 13,14 | B,M | 0.060 |  | X | X |
| **NS SU 290** | F | 41-50 | 23,24  25  26 | L,B  L,B,M  B,D | 2.314 | X | X | X |
| **NS SU 292 Aa** | M | >50 | 22  23,24,25 | L  L,B | 0.115 | X | X | X |
| **NS SU 293-306** | M | 31-40 | 41,42,43  45,46,47  44 | L  B,M  D | 0.185 | X | X | X |
| **NS SU 299 Sa** | IND | 1-6 | 41,43,44 | L,B | 0.022 |  | X |  |
| **NS SU 301 Ab** | M | 20-25 | 13,16 | L,B | 0.023 |  | X |  |
| **NS SU 302 Aa** | F | 41-50 | 45,46,47,48 | L,B | 0.092 | X | X | X |
| **NS SU 302 Ab** | F | 19-30 | 42,44 | L,B | 0.064 |  | X | X |
| **NS SU 304 Aa** | F | 31-40 | 11,12 | L | 0.012 |  |  | X |
| **NS SU 307** | F | 31-40 | 21,23 | L,B,M | 0.096 | X | X | X |
| **NS SU 308** | F | 19-30 | 23,24 | B,M | 0.023 |  | X |  |
| **NS SU 309 Aa** | F | 41-50 | 25,26  43,45,46 | B  L,B | 0.112 | X | X | X |
| **NS SU 310 Aa** | F | 31-40 | 41,42,43  46,47,48 | L,B  B | 0.85 | X | X | X |
| **NS SU 311 Aa** | M | 31-40 | 41,42,43  46,47,48  13,14,15 | L,B  B  B,L,M | 0.120 | X | X | X |
| **NS SU 316 Aa** | M | 13-18 | 35,36,37 | L,B | 0.050 |  | X | X |
| **NS SU 317 Aa** | M | >50 | 43  46,47 | L,B,D  L,B | 0.024 |  | X |  |
| **NS SU 318 Aa** | M | 19-30 | 41,42,43,45  46,47,48  22,23 | L,B  B  L | 0.107 | X | X | X |
| **NS SU 319** | F | 19-30 | 11,12,13,  14,15  46,47,48 | L,B  D,M  B | 0.0195 | X | X | X |
| **NS SU 320** | ND | 13-18 | 17,18 | L,B | 0.022 |  | X |  |
| **NS SU 321** | M | 51-60 | 23,24,25  31,32,33  36,37 | L,B  L  B | 0.391 | X | X | X |
| **NS SU 322** | F | 31-40 | 32,33,36  44,45  17,18 | L  B  B | 0.090 | X | X | X |
| **NS SU 325** | ND | GA | 13,16 | L,B | 0.042 |  | X | X |
| **NS SU 326 Aa** | F | 31-40 | 42,44,46  11,15,17  12,43,16  31,32 | L,B  B  M  L | 0.212 | X | X | X |
| **NS SU 327 Sa** | F | 13-18 | 11,12  21,22  16,26,  36,46 | B  B  L,B  B | 0.084 | X | X | X |
| **NS US 328 Ab** | ND | GA | 11,17,23,34 | L,B | 0.054 | X |  |  |
| **NS SU 331 Aa** | M | 31-40 | 21,24 | L,B | 0.018 |  | X |  |
| **NS SU 333** | IND | 7-12 | 25,26,27 | L,B | 0.023 |  | X |  |
| **NS SU 336 Aa** | M | 31-40 | 44,45 | B | 0.16 |  |  | X |
| **NS SU 341 Aa** | M | 13-18 | 32,33  36,37,38 | L  L,B | 0.080 | X | X | X |
| **NS SU 344 Aa** | M | 19-30 | 44,45,46,47 | B | 0.055 |  | X | X |
| **NS SU 346 Ab** | F | 41-50 | 14,15  41 | L,B  L | 0.041 |  | X | X |
| **NS SU 347 Ab** | ND | 19-30 | 11,12,13  21,22,23 | L,B  L,B | 0.130 | X | X | X |
| **NS SU 351 Sb** | IND | 7-12 | 21 | B,D | 0.021 |  | X |  |
| **NS SU 356** | ND | GA | 17,18  25 | B  L | 0.102 | X | X | X |
