# Supplementary material for: A multidisciplinary approach for investigating dietary and medicinal habits of the Medieval population of Santa Severa (7th-15th centuries, Rome, Italy)
Source: PLoS One. 2020 Jan 28;15(1):e0227433. doi: 10.1371/journal.pone.0227433 (PMC6986732; doi:10.1371/journal.pone.0227433)
Supplement: S2 Table — (DOCX) [file pone.0227433.s002.docx]

**S2 Table.** Light microscopy results of the laboratory contamination tests.

| **Context** | **Location of traps (number)** | **Starches** | **Pollen grains** | **Other*** | **Total** |
| --- | --- | --- | --- | --- | --- |
| **Work day** | workbench (10) | 351 | 12 | 19 | **382** |
|  | floor (10) | 219 | 47 | 151 | **417** |
|  | shelf (5) | 59 | 24 | 1 | **84** |
|  | hood (5) | 4 | 7 | 4 | **15** |
| **After decontamination** | workbench (10) | 1 | 0 | 2 | **3** |
|  | floor (10) | 9 | 0 | 2 | **11** |
|  | shelf (5) | 0 | 1 | 0 | **1** |
|  | hood (5) | 0 | 0 | 1 | **1** |

*hairs, fibres, dust residues
