# Supplementary material for: A multidisciplinary approach for investigating dietary and medicinal habits of the Medieval population of Santa Severa (7th-15th centuries, Rome, Italy)
Source: PLoS One. 2020 Jan 28;15(1):e0227433. doi: 10.1371/journal.pone.0227433 (PMC6986732; doi:10.1371/journal.pone.0227433)
Supplement: S3 Table — (DOCX) [file pone.0227433.s003.docx]

| **Individual** | **Starches** | **Ovoid and partially gelatinized starches** | **Indeterminate particles** | **Pollen grains** | **Fungal spores/hyphae** | **Plant fibres** |
| --- | --- | --- | --- | --- | --- | --- |
| **NS SU 78 Aa** |  | 1 | 2 |  |  | 1 |
| **NS SU 95 Aa** |  |  | 1 | 1 |  |  |
| **NS SU 138 Ab** | 5 |  |  |  | 1 |  |
| **NS SU 168** |  | 1 |  |  |  |  |
| **NS SU 196 Aa** |  |  |  |  | 4 | 1 |
| **NS SU 202** |  |  |  |  |  |  |
| **NS SU 293-306** |  |  | 3 |  | 1 |  |
| **NS SU 311 Aa** |  |  |  | 2 | 1 |  |
| **NS SU 321** |  |  |  |  |  |  |
| **NS SU 356** |  | 6 |  |  |  |  |

**S3 Table.** Light microscopy results obtained from the washing water applied on ancient dental calculus before the cleaning procedure.
