# Supplementary material for: A multidisciplinary approach for investigating dietary and medicinal habits of the Medieval population of Santa Severa (7th-15th centuries, Rome, Italy)
Source: PLoS One. 2020 Jan 28;15(1):e0227433. doi: 10.1371/journal.pone.0227433 (PMC6986732; doi:10.1371/journal.pone.0227433)
Supplement: S4 Table — In addition, length of PCR amplicons and annealing temperatures were also reported. (DOCX) [file pone.0227433.s004.docx]

**S4 Table.** The primers used for “Spike PCR” (L15996 e H16401) and detection of aDNA relative to bovine (Bov84/90-F/R), pig (Sus85-F/R; Sus98-F/R), ovine (Ovis-F/R), chicken (Gall-F/R) and fishes (Fish_miniA_F/R; Fish_miniC_F/R) were shown. In addition, length of PCR amplicons and annealing temperatures were also reported.

| **Primer** | **Sequence** | **Product length** | **Ta** |
| --- | --- | --- | --- |
| L15996 | CGAAGCTTCTCCACCATTAGCACCCAAAG | 405 bp | 60 °C |
| H16401 | GCGGGATATTGATTTCACGG |  |  |
| Bov84-F | TTAGTTGAATTAGGCCATGAAGCA | 84 bp | 54 °C |
| Bov84/90-R | GTTTAAATAGGGTTAAGATGCACTCAATC |  |  |
| Bov90-F | GAGTGCTTAGTTGAATTAGGCC | 90 bp | 54 °C |
| Bov84/90-R | GTTTAAATAGGGTTAAGATGCACTCAATC |  |  |
| Sus85-F | GGAGCAGTGTTCGCCATTAT | 85 bp | 57 °C |
| Sus85-R | TTTTTGCTCATGCTTGGTTG |  |  |
| Sus98-F | GCGGGTACTGGATGAACTGT | 98 bp | 57 °C |
| Sus98-R | CCTGCAAGGTGTAGGGAGAA |  |  |
| Ovis-F | CCCTAGGTTTCATCTTTCTTTTCACA | 67 bp | 60 °C |
| Ovis-R | AGGGAGGAGTTGGCTAGAACAA |  |  |
| Gall-F | AACCTCCTCCAGCGGATAATAAT | 66 bp | 59 °C |
| Gall-R | TTTGTTGGTGGCTGCTTGAA |  |  |
| Fish_miniA_F | CACGACGTTGTAAAACGACACIAAICAIAAAGAYATYGGC | 129 bp | 46 °C |
| Fish_miniA_R | GGATAACAATTTCACACAGGAARAAAATYATAACRAAIGCRTGIGC |  |  |
| Fish_miniC_F | CACGACGTTGTAAAACGACACYAAICAYAAAGAYATIGGCAC | 127 bp | 46 °C |
| Fish_miniC_R | GGATAACAATTTCACACAGGGAARATCATAATGAAGGCATGIGC |  |  |
