# Supplementary material for: A multidisciplinary approach for investigating dietary and medicinal habits of the Medieval population of Santa Severa (7th-15th centuries, Rome, Italy)
Source: PLoS One. 2020 Jan 28;15(1):e0227433. doi: 10.1371/journal.pone.0227433 (PMC6986732; doi:10.1371/journal.pone.0227433)
Supplement: S5 Table — (DOCX) [file pone.0227433.s005.docx]

| **NS SU 27 Ac** | |  |
| --- | --- | --- |
| Sugars | Lactose | |
| Amino acids | Serine | |
| Fatty acids | 9-Octadecenoic acid  Eicosapentaenoic acid  Octanoic acid  Tetradecanoic acid  Undecanoic acid | |
| Alcohols | Kaurenol  Falcarinol | |
| Vitamins | Ascorbic acid | |
| Terpens and derivatives | alpha-Bisabolene  alpha-Bisabolol  Cadina-3,9-diene  Carveol  Cedra-diol  Dihydroartemisinin  Ledene  Limonenol  Myrtenol | |
| Phenolic compounds and derivatives | Anisole  Coumarin  Hydrocinnamic acid | |

| **NS SU 78 Aa** | |
| --- | --- |
| Sugars | Mannitol |
| Amino acids | Serine  Valine |
| Fatty acids | 10-Octadecenoic acid  5-Heptenoic acid  7-Hexadecenoic acid  9,12-Octadecadienoic acid  Heptacosanoic acid  Hexadecanoic acid  Octadecanoic acid  Undecanoic acid |
| Alcohols | 1-Hexadecanol  Pentadecen-1-ol |
| Terpens and derivatives | alpha-Cubebene  beta-Carotene  Bisabolene  Farnesol  Ocimene  Pseduosarsasapogenin |
| Phenolic compounds and derivatives | Benzeneacetic acid  Cinnamic acid  Coumarin  Caffeic acid |
| Steroidal compounds | Cardenolide  Cholesterol  Stigmasterol |
| Others vegetal and non-vegetal marker | Agaricic acid |

| **NS SU 78 Ag** | |
| --- | --- |
| Sugars | Lactose  Sedoheptulose |
| Amino acids | Alanine |
| Fatty acids | 10-Octadecenoic acid  13-Docosenoic acid  9-Octadecenoic acid  Butanoic acid  Decanoic acid  Docosahexanoic acid  Octadecanoic acid  Tridecanoic acid  Undecanoic acid |
| Vitamins | Tocopherol |
| Alcaloids and derivatives | Acetyl betonicine |
| Terpens and derivatives | alpha-Bisabolene  alpha-Pinene  beta-Carotene  Cymene  Kaurenol  Limonenol  Pseduosarsasapogenin |
| Phenolic compounds and derivatives | Butylcatechol  Benzoic acid |

| **NS SU 95 Aa** | |
| --- | --- |
| Sugars | Glucitol |
| Amino acids | Alanine  Lysine  Valine |
| Fatty acids | 10-Octadecenoic acid  9,12-Octadecadienoic acid  Butanoic acid  Dodecanoic acid  Eicosatrienoic acid  Heptanoic acid  Heptacosanoic acid  Hexanoic acid  Octadecanoic acid  Octadecanoic acid  Octanoic acid  Palmitic acid  Pentanoic acid  Tetradecanoic acid  Tridecanoic acid |
| Alcohols | 1-Heptanol  3-Hexadecanol |
| Terpens and derivatives | alpha-Bisabolene  Cadinol  Citronellol  Digitoxin  Emicymarin  Geranylgeraniol  Globulol  Isopinocarveol  Limonenol  Spathulenol |
| Phenolic compounds and derivatives | Homogentisic acid  Dihydrocoumarin  Cinnamic acid |
| Steroidal compounds | Cholest-5-en-3-ol |

| **NS SU 99** | |
| --- | --- |
| Sugars | Sorbitol |
| Amino acids | Alanine |
| Fatty acids | 9,12-Octadecadienoic acid  9-Octadecenoic acid  alpha-Linolenic acid  Eicosatrienoic acid  Octadecanoic acid  Triacontanoic acid |
| Alcohols | 1-Decanol  Tetracosanol-1 |
| Terpens and derivatives | alpha-Irone  beta-Carotene  Caranol  Cedrandiol  Cucurbitacin b  Digitoxin  Geranylgeraniol  Isopulegol  Thymol |
| Phenolic compounds and derivatives | Cinnamic acid  Methanoazulene |
| Steroidal compounds | Sitosterol  Cholestadienol |
| Others vegetal and non-vegetal marker | Glucobrassicin  Glutaric acid |

| **NS SU 104 Ab** | |
| --- | --- |
| Sugars | Lactose |
| Amino acids | Alanine |
| Fatty acids | 13-Docosenoic acid  9,12-Octadecadienoic acid  9-Octadecenoic acid  Eicosapentaenoic acid  Heptacosanoic acid  Triacontanoic acid  Tridecanoic acid |
| Terpens and derivatives | alpha-Limonene  alpha-Pinene  beta-Carotene  Aromandendrene  Bergamotol  Longiborneol |
| Phenolic compounds and derivatives | Pyrocatechol  Pyrogallol |
| Steroidal compounds | beta-Sitosterol  Cholestenol |

| **NS SU 115** | |
| --- | --- |
| Sugars | Rhamnitol |
| Amino acids | Alanine  Threonine |
| Fatty acids | 9,12-Octadecadienoic acid  Pentenoic acid  Butanoic acid  Eicosapentaenoic acid  Heptacosanoic acid |
| Alcohols | Kaurenol |
| Terpens and derivatives | 1,2-Dihydrolinalool  alpha-Bisabolene  beta -Carotene  tau-Cadinol  Dihydroartemisinin  Emicymarin  Isolongifolene  Ocimene  Menthadienol  Myrtenol |
| Phenolic compounds and derivatives | Cyclopentanocoumarine  Dihydrocoumarin |
| Steroidal compounds | Dibromostigmasterol  Ergostenol |
| Others vegetal and non-vegetal marker | Dehydroelsholtzia ketone |

| **NS SU 124 Aa** | |
| --- | --- |
| Sugars | Galactitol  Galacto-heptulose  Lactose  Rhamnitol |
| Amino acids | Alanine |
| Fatty acids | 11,14-Eicosadienoic acid  11-Octadecenoic acid  9,12-Octadecadienoic acid  9-Octadecenoic acid  Butanoic acid  Decanoic acid  Eicosanoic acid  Eicosapentaenoic acid  Eicosatrienoic acid  Heptacosanoic acid  Hexadecanoic acid  Octadecanoic acid  Pentanoic acid |
| Alcohols | 3-Dodecanol |
| Vitamins | Ascorbic acid  Nicotinamide |
| Alcaloids and derivatives | Pseudopelletierine |
| Terpens and derivatives | beta-Ionone  Aromadendranediol  Eudesmol  Limonene  Myrtenol  Patchoulane  Perilla alcohol angelate  Sesquisabinene  Thymol  Zingiberenol |
| Phenolic compounds and derivatives | Benzoic acid  Homogentisic acid  Hydrocinnamic acid  Scoparone |
| Steroidal compounds | beta-Sitosterol  Cholestenol  Cholesterol |
| Combustion marker | Naphthalene  Phenanthrene |

| **NS SU 129 Aa** | |
| --- | --- |
| Sugars | Lactose  Sedoheptulose |
| Amino acids | Alanine  Valine |
| Fatty acids | 7-Hexadecenoic acid  Butanoic acid  Dodecanoic acid  Hexadecanoic acid  Nonanoic acid  Octadecanoic acid  Tetradecanoic acid  Tridecanoic acid |
| Alcohols | 1-Decanol  Kaurenol |
| Alcaloids and derivatives | Reticuline |
| Terpens and derivatives | Farnesene  Geranylgeraniol  Isoborneol  Pseduosarsasapogenin  Strophanthidol |
| Phenolic compounds and derivatives | Coumarin  Dihydrocoumarin |
| Steroidal compounds | Brassicasterol  Campesterol  Cardenolide  Stigmasterol |
| Others vegetal and non-vegetal marker | Lupetidine  Lutidine |

| **NS SU 129 Ac** | |
| --- | --- |
| Sugars | Galactopyranose  Glucitol  Lactose  Rhamnitol |
| Fatty acids | 9,12-Octadecadienoic acid  Docosahexanoic acid  Dodecanoic acid  Eicosapentaenoic acid  Heptacosanoic acid  Hexadecatrienoic acid  Octadecanoic acid |
| Alcohols | 1-Octanol  Nonanol |
| Terpens and derivatives | alpha-Bisabolene  Cedrandiol  Pseduosarsasapogenin |
| Phenolic compounds and derivatives | Dihydrocoumarin  Octahydrocoumarin |
| Steroidal compounds | Brassicasterol |
| Others vegetal and non-vegetal marker | Lupetidine |
| Combustion marker | 1-(Toluene-4-sulfonyl) azetidine-2-carboxylic acid |

| **NS SU 137 Ab** | |
| --- | --- |
| Sugars | Galactose  Mannitol |
| Fatty acids | Butanoic acid  Eicosapentaenoic acid  Pentadecanoic acid  Tetradecanoic acid |
| Alcohols | 1-heptanol |
| Terpens and derivatives | alpha-Bisabolol  Pseduosarsasapogenin |
| Steroidal compounds | Campesterol  Stigmasterol |
| Phenolic compounds and derivatives | Cyclopentanocoumarine |

| **NS SU 150** | |
| --- | --- |
| Sugars | Glucitol  Rhamnitol |
| Amino acids | Alanine |
| Fatty acids | 9,12-Octadecadienoic acid  9-Octadecenoic acid  Eicosapentaenoic acid  Heptacosanoic acid  Pentanoic acid  Pentenoic acid |
| Alcohols | Falcarinol |
| Vitamins | Tocopherol |
| Terpens and derivatives | Cadinol  Carene  Limonenol  Thymol |
| Phenolic compounds and derivatives | Methanoazulene  Cyclopentanocoumarine  Coumarin  Dihydrocoumarin |
| Steroidal compounds | Fucosterol  Stigmasterol |

| **NS SU 151 Aa** | |
| --- | --- |
| Sugars | Galactitol  Glucose |
| Amino acids | Alanine |
| Fatty acids | 10-Octadecenoic acid  Docosahexanoic acid  Hexadecanoic acid |
| Alcohols | 5-Octenol  Kauranolo |
| Terpens and derivatives | alpha-Atlantone  beta-Phellandrene  Eucalyptol  Kauranal  Ledene oxide  Myrtanol  Neoisolongifolene  Pinanediol  Pseduosarsasapogenin  Spathulenol  Ursenol  Verbenone  Verrucarol |
| Phenolic compounds and derivatives | Benzenepropanoic acid  Dihydrocoumarin |
| Others vegetal and non-vegetal marker | Glucobrassicin |
| Combustion marker | Naphthalene  Phenanthrene |

| **NS SU 154 Aa** | |
| --- | --- |
| Sugars | Galactitol |
| Fatty acids | 9,12-octadecadienoate |
| Terpens and derivatives | Nerolidol  Sesquiphellandrene |
| Phenolic compounds and derivatives | Hydrocinnamic acid |
| Steroidal compounds | Cholestenol |
| Others vegetal and non-vegetal marker | Lupetidine |

| **NS SU 154 Ab** | |
| --- | --- |
| Sugars | Rhamnose |
| Amino acids | Alanine |
| Fatty acids | 9,12-Octadecadienoic acid  9-Octadecenoic acid  Hexadecanoic acid  Triacontanoic acid |
| Alcohols | Falcarinol |
| Terpens and derivatives | alpha-Bisabolene  alpha-Pinene  beta-Carotene  beta-copaene  gamma-Elemene  Cymenol  Geranylgeraniol  Limonene |
| Phenolic compounds and derivatives | Cinnamic acid  Pyrogallol |
| Steroidal compounds | beta-Sitosterol  Cholestenol |

| **NS SU 155** | |
| --- | --- |
| Sugars | Fructose  Trehalose |
| Fatty acids | Hexadecanoic acid  Octadecanoic acid |
| Terpens and derivatives | alpha-Farnesene  beta Carotene  Artemiseole  Carvacrol  Cycloartanol  Eudesmenol  Isoborneol  Sesquisabinene  Squalene  Thymol |

| **NS SU 157 Aa** | |
| --- | --- |
| Sugars | Rhamnitol |
| Fatty acids | 10-Octadecenoic acid  Docosahexanoic acid |
| Terpens and derivatives | alpha-Bisabolene  alpha-Limonene  Verbenone |
| Phenolic compounds and derivatives | Cyclopropazulenol  Dihydrocoumarin  Hydrocinnamic acid |
| Steroidal compounds | Cholestenol  Lanostenol |

| **NS SU 158 Aa** | |
| --- | --- |
| Sugars | Lactose  Glucitol |
| Amino acids | Alanine  Serine |
| Fatty acids | 2,3-Dihydroxyoctadecanoic acid  9-Octadecenoic acid  Decanoic acid |
| Alcohols | Tetracosanol |
| Terpens and derivatives | delta-Selinene  Linalool |
| Phenolic compounds and derivatives | Cyclopropazulenol |
| Steroidal compounds | beta-Sitosterol  Cholestenol |
| Others vegetal and non-vegetal marker | Mustard oil |

| **NS SU 163 Ab** | |
| --- | --- |
| Sugars | Lactose  Mannitol |
| Amino acids | Alanine |
| Fatty acids | 9,12-Octadecadienoic acid  9-Octadecenoic acid  Eicosapentaenoic acid  Hexacosanoic acid  Hexadecanoic acid  Tridecanoic acid |
| Terpens and derivatives | beta-Carotene  beta-Irone  delta-Selinene  Eudesmadienol  Isolongifolene  Pseduosarsasapogenin |
| Phenolic compounds and derivatives | Homogentisic acid |
| Steroidal compounds | beta-Sitosterol  Cholestenol |

| **NS SU 164 Ab** | |
| --- | --- |
| Sugars | Galactitol  Lactose |
| Fatty acids | 13-Docosenoic acid  7-Hexadecenoic acid  Butanoic acid  Decanoic acid  Dodecanoic acid  Tridecanoic acid |
| Alcohols | 3-Dodecanol  Falcarinol |
| Vitamins | Tocopherol |
| Terpens and derivatives | alpha-Bisabolene  beta-Linalool  Cedrandiol  Farnesol  Pseduosarsasapogenin  Spathulenol |
| Phenolic compounds and derivatives | Dihydrocoumarin  Estragole  Ethenoazulene |
| Steroidal compounds | Cycloergostenol |

| **NS SU 165** | |
| --- | --- |
| Sugars | Rhamnitol |
| Amino acids | Alanine  Valine |
| Fatty acids | 3-Pentenoic acid  Decanoic acid  Dodecanoic acid  Heptadecanoic acid  Octanoic acid  Undecanoic acid |
| Alcohols | Falcarinol |
| Terpens and derivatives | alpha-Bisabolene  alpha-Copaene  Citronellol  Eudesmenol  Patchoulane |
| Steroidal compounds | Dibromostigmasterol |
| Others vegetal and non-vegetal marker | alpha-Ergosine |

| **NS SU 177 Ab** | |
| --- | --- |
| Fatty acids | 7-Hexadecenoic acid  Eicosapentaenoic acid  Heptacosanoic acid |
| Alcaloids and derivatives | Picrotoxin |
| Terpens and derivatives | alpha-Ylangene  Limonenol  Longipinocarveol  Pseduosarsasapogenin  Squalane |
| Phenolic compounds and derivatives | Homogentisic acid  Hydrocinnamic acid  Resorcinol |
| Steroidal compounds | Brassicasterol  Dibromostigmasterol |
| Others vegetal and non-vegetal marker | Isothiocyanate |
| Combustion marker | Decalin |

| **NS SU 179 Aa** | |
| --- | --- |
| Sugars | Galactitol  Glucitol |
| Amino acids | Alanine |
| Fatty acids | 9-Octadecenoic acid  Butanoic acid  Decanoic acid  Docosahexanoic acid  Hexadecanoic acid  Hexanoic acid  Pentadecanoic acid |
| Alcohols | Falcarinol |
| Terpens and derivatives | alpha-Pinene  Caryophyllene  Cedrandiol  Isocitronellol  Limonenol  Linalool  Verbenone |
| Phenolic compounds and derivatives | Dihydrocoumarin  Homogentisic acid  Pyrogallol |

| **NS SU 190 Aa** | |
| --- | --- |
| Sugars | Galactose |
| Fatty acids | Hexadecanoic acid  Pentanoic acid |

| **NS SU 190 Ad** | |
| --- | --- |
| Sugars | Glucopyranoside |
| Amino acids | Alanine |
| Fatty acids | Decanoic acid  Eicosadienoic acid  Eicosatrienoic acid  Hexadecanoic acid  Octadecanoic acid  Tridecanoic acid |
| Alcohols | Kaurenol |
| Vitamins | Tocopherol |
| Terpens and derivatives | Khusimone  Myrtenol  Ocimene  Pseduosarsasapogenin  Thunbergol |

| **NS SU 192** | |  |
| --- | --- | --- |
| Amino acids | Alanine | |
| Fatty acids | 9,12-Octadecadienoic acid  9-Hexadecenoic acid  9-Octadecenoic acid  Butanoic acid  Docosahexanoic acid  Hexadecanoic acid  Tetracosanoic acid | |
| Phenolic compounds and derivatives | Cumene | |
| Steroidal compuonds | Brassicasterol  Cholestenol | |
| Others vegetal and non-vegetal marker | Tartaric acid | |

| **NS SU 196 Aa** | |
| --- | --- |
| Fatty acids | Heptacosanoic acid  Pentanoic acid |
| Terpens and derivatives | Thymol |

| **NS SU 202** | |
| --- | --- |
| Amino acids | Alanine  Serine |
| Fatty acids | Eicosatrienoic acid  Heptacosanoic acid  Pentanoic acid |
| Terpens and derivatives | Limonene  Linalool |

| **NS SU 205 Ac** | |
| --- | --- |
| Sugars | Lactose |
| Fatty acids | 11,14-Eicosadienoic acid  Pentadecanoic acid  Undecanoic acid |
| Vitamins | Tocopherol |
| Steroidal compounds | Dibromostigmasterol |
| Phenolic compounds and derivatives | Ethenoazulene |

| **NS SU 210** | |  |
| --- | --- | --- |
| Fatty acids | 9,12-Octadecadienoic acid  9-Octadecenoic acid  13-Docosenoic acid  Docosahexanoic acid  Heptacosanoic acid  Hexadecanoic acid  Pentadecanoic acid  Tetracosanoic acid | |
| Phenolic compounds and derivatives | Methanoazulene  Dihydrocoumarin | |
| Steroidal compounds | beta-Sitosterol  Cholestenol | |

| **NS SU 215** | |
| --- | --- |
| Sugars | Galactitol |
| Fatty acids | 13-Octadecenoic acid  7-Hexadecenoic acid  Dodecanoic acid  Tridecanoic acid |
| Alcohols | Falcarinol  Kaurenol |
| Vitamins | Tocopherol |
| Terpens and derivatives | alpha-Bisabolene  Geranylgeraniol  Limonenol  Thymol |
| Steroidal compounds | Dibromostigmasterol |

| **NS SU 217 Aa** | |
| --- | --- |
| Sugars | Galacto-heptulose  Inositol |
| Amino acids | Alanine  Citrulline |
| Fatty acids | Eicosatrienoic acid  9,12-Octadecadienoic acid  9-Octadecenoic acid  Butanoic acid  Docosahexanoic acid  Eicosanoic acid  Heptanoic acid  Hexadecanoic acid  Octadecanoic acid  Triacontanoic acid  Tridecanoic acid |
| Alcohols | Falcarinol |
| Vitamins | Ascorbic acid |
| Terpens and derivatives | delta-Selinene  Cumic alcohol  Digitoxin  Limonenol |
| Phenolic compounds and derivatives | Homogentisic acid |
| Steroidal compounds | Ergost-5-en-3-ol  Stigmastadienol  Cholestenol |
| Others vegetal and non-vegetal marker | Glucobrassicin  Succinic acid |

| **NS SU 219 Aa** | |
| --- | --- |
| Sugars | Arabinitol  Galactose  Lactose |
| Amino acids | Alanine |
| Fatty acids | 9,12-Octadecadienoic acid  9-Octadecenoic acid  Heptacosanoic acid |
| Alcohols | Tetracosanol |
| Terpens and derivatives | Limonene |
| Others vegetal and non-vegetal marker | Malic acid |

| **NS SU 221 Aa** | |
| --- | --- |
| Sugars | Glucitol  Glucose  Inositol |
| Amino acids | Isoasparagine |
| Fatty acids | 9-Octadecenoic acid  Eicosapentaenoic acid  Eicosatrienoic acid  Octadecanoic acid  Pentanoic acid  Triacontanoic acid |
| Alcohols | Octanol  Tetracosanol |
| Terpens and derivatives | Bergamiol  Carvone  Cycloartanol  Levoverbenone  Limonenol |
| Phenolic compounds and derivatives | Dihydrocoumarin |
| Steroidal compounds | Cholestadienol |
| Others vegetal and non-vegetal marker | Furanone, 5-butyldihydro-4-methyl  Tartaric acid |

| **NS SU 231** | |
| --- | --- |
| Sugars | Inositol  Sedoheptulose |
| Amino acids | Lysine  Serine |
| Fatty acids | 11-Octadecenoic acid  13-Docosenoic acid  7-Hexadecenoic acid  9,12-Octadecadienoic acid  9-Octadecenoic acid  Docosahexanoic acid  Eicosanoic acid  Eicosatrienoic acid  Heptacosanoic acid  Hexadecanoic acid  Malic acid  Octadecanoic acid  Pentanoic acid  Tridecanoic acid |
| Alcohols | 1-Heptanol  1-Hexanol  1-Octanol  Tetracosanol  Piperonyl alcohol |
| Terpens and derivatives | Citronellol  Limonene |
| Phenolic compounds and derivatives | Benzoic acid  Hydrocinnamic acid |
| Others vegetal and non-vegetal marker | Acetovanillone |

| **NS SU 235** | |
| --- | --- |
| Amino acids | Leucine |
| Fatty acids | 9,12-Octadecadienoic acid  Heptacosanoic acid  Pentanoic acid |
| Others vegetal and non-vegetal marker | Glucobrassicin |

| **NS SU 240 Aa** | |
| --- | --- |
| Sugars | Inositol |
| Fatty acids | 9,12-Octadecadienoic acid  9-Octadecenoic acid  Decanoic acid  Heptacosanoic acid  Octadecanoic acid |
| Alcohols | 3-Dodecanol  Tetracosanol |
| Phenolic compounds and derivatives | Octahydrocoumarin  Cyclopentanocoumarine  Homogentisic acid |
| Steroidal compounds | Cholesterol |

| **NS SU 241** | |
| --- | --- |
| Amino acids | Valine |
| Fatty acids | 11-Octadecenoic acid  9,12-Octadecadienoic acid  9-Octadecenoic acid  Butanoic acid  Eicosanoic acid  Heptacosanoic acid  Hexadecanoic acid  Hexanoic acid  Octadecanoic acid  Tetradecanoic acid |
| Terpens and derivatives | Ocimenol |
| Phenolic compounds and derivatives | Coumarin |
| Others vegetal and non-vegetal marker | gamma-Octalactone  Malic acid |

| **NS SU 283 Aa** | |
| --- | --- |
| Sugars | Galactopyranose  Lactose  Mannitol |
| Amino acids | Lysine  Serine  Valine |
| Fatty acids | 11,14-Eicosadienoic acid  9-Octadecenoic acid  Decanoic acid  Octadecanoic acid  Pentadecanoic acid  Pentanoic acid |
| Alcohols | Piperonyl alcohol  Tetracosanol |
| Phenolic compounds and derivatives | Homogentisic acid  Pyrocatechol |
| Others vegetal and non-vegetal marker | Artemisia ketone  Glucobrassicin  Succinic acid |

| **NS SU 284 Aa** | |
| --- | --- |
| Amino acids | Alanine  Isoasparagine  Lysine |
| Fatty acids | 9,12-Octadecadienoic acid  9-Octadecenoic acid  Octadecanoic acid  Undecanoic acid |
| Terpens and derivatives | Carvacrol  Ocimenol |
| Others vegetal and non-vegetal marker | Acetovanillone  Succinic acid |

| **NS SU 287 Aa** | | |
| --- | --- | --- |
| Sugars | | Lactose |
| Amino acids | | Alanine |
| Fatty acids | | 9-Octadecenoic acid  Butanoic acid  Decanoic acid  Heptacosanoic acid  Octadecanoic acid  Tetradecanoic acid |
| Alcohols | | 3-Nonenol  4-Dodecanol |
| Terpens and derivatives |  | Squalene |
| Phenolic compounds and derivatives | | 1,2-Benzenediol |
| Others vegetal and non-vegetal marker | | Artemisia ketone |

| **NS SU 287 Ab** | |
| --- | --- |
| Sugars | Arabinose  Galactopyranose  Inositol |
| Amino acids | Alanine  Asparagine  Glutamic acid  Serine |
| Fatty acids | 9-Octadecenoic acid  Decanoic acid  Octadecanoic acid  Pentanoic acid |
| Alcohols | 1,3-Cyclohexanediol  2-Butanol  Cyclodecanol  Piperonyl alcohol |
| Terpens and derivatives | 1,2-Dihydrolinalool  Thymol |
| Phenolic compounds and derivatives | Homogentisic acid  Pyrogallol |
| Steroidal compounds | Cholesterol |
| Others vegetal and non-vegetal marker | Furanone, dihydro-5-propyl-  Succinic acid |

| **NS SU 287 Ac** | |
| --- | --- |
| Sugars | Lactose |
| Amino acids | Norleucine  Serine |
| Fatty acids | 13-Docosenoic acid  9,12-Octadecadienoic acid  9-Octadecenoic acid  Eicosatrienoic acid  Octadecanoic acid  Pentanoic acid |
| Alcohols | 3-Nonen-1-ol  Tetracosanol |
| Terpens and derivatives | Citronellol  Ocimenol |
| Phenolic compounds and derivatives | Benzoic acid  Cyclopentanocoumarine  Gentisic acid  Homogentisic acid |
| Others vegetal and non-vegetal marker | Artemisia ketone  Furanone, dihydro-5-methyl-  Glucobrassicin |

| **NS SU 289** | |
| --- | --- |
| Sugars | Galactosan  Rhamnitol |
| Amino acids | Alanine  Serine |
| Fatty acids | 9-Octadecenoic acid  Butanoic acid  Cyclopentaneundecanoic acid  Decanoic acid  Dodecanoic acid  Eicosanoic acid  Heptacosanoic acid  Hexadecanoic acid  Octadecanoic acid  Octanoic acid  Pentanoic acid  Tetradecanoic acid |
| Alcohols | 1-Heptanol  3-Dodecanol  Tetracosanol |
| Terpens and derivatives | Cycloartanol  Dihydrocitronellol  Squalene |
| Phenolic compounds and derivatives | Benzoic acid  Cyclopentanocoumarine  Homogentisic acid |
| Steroidal compounds | beta-Sitosterol  Cholesterol |
| Others vegetal and non-vegetal marker | Acetovanillone  Artemisia ketone  Furanone, 5-ethyldihydro  Glucobrassicin  Succinic acid |

| **NS SU 290** | |
| --- | --- |
| Sugars | Galactosan  Galactose  Glucose  Mannose |
| Amino acids | Alloisoleucine  Serine |
| Fatty acids | 11-Octadecenoic acid  5-Valeric acid  7-Hexadecenoic acid  9,12-Octadecadienoic acid  Glutaric acid  Heptacosanoic acid  Hexadecanoic acid  Octadecanoic acid  Octanoic acid  Palmitic acid  Pentadecanoic acid  Tetradecanoic acid  Tridecanoic acid  Undecanoic acid |
| Alcohols | 1-Hexanol  1-Heptanol  1-Octen-3-ol |
| Vitamins | alpha-Tocopherolmannoside  Nicotinic acid |
| Terpens and derivatives | 1,2-Dihydrolinalool  Bergamiol  beta-Carotene  beta-Linalool  Cycloisolongifolene  Deoxyartemisinin  Limonene |
| Phenolic compounds and derivatives | Benzoic acid  Cyclopentanocoumarine  Resorcinol |
| Steroidal compounds | Sitosterol |
| Others vegetal and non-vegetal marker | Cinnamic acid |

| **NS SU 292 Aa** | |
| --- | --- |
| Sugars | Galactitol  Rhamnose |
| Amino acids | Alanine  Lysine |
| Fatty acids | 11,14-Eicosadienoic acid  9,12-Octadecadienoic acid  9-Octadecenoic acid  Decanoic acid  Docosahexanoic acid  Eicosanoic acid  Heptacosanoic acid  Octadecanoic acid  Pentanoic acid  Triacontanoic acid |
| Alcohols | 1-Dodecanol  1-Octanol  Tetracosanol |
| Vitamins | Vitamin A |
| Terpens and derivatives | Artemisia alcohol  Carvacrol  Limonenol  Thymol |
| Phenolic compounds and derivatives | Catechol  Cyclopentacoumarin  Gallic acid  Homogentisic acid  Pyrogallol |
| Steroidal compounds | beta-Sitosterol  Cholest-8-en-3-ol |
| Others vegetal and non-vegetal marker | Malic acid |
| Combustion marker | Toluene |

| **NS SU 293-306** | |
| --- | --- |
| Sugars | Sedoheptulose |
| Amino acids | Proline |
| Fatty acids | 9,12-Octadecadienoic acid  9-Octadecenoic acid  Docosanoic acid  Eicosanoic acid  Eicosapentaenoic acid  Heptacosanoic acid  Hexadecanoic acid  Octadecanoic acid  Pentadecanoic acid  Tetradecanoic acid |
| Alcohols | 1-Octanol |
| Terpens and derivatives | Pseduosarsasapogenin  Tetrahydrolinalool |
| Phenolic compounds and derivatives | Benzoic acid  Cyclopentanocoumarine  Homogentisic acid |
| Steroidal compounds | Cholest-8-en-3-ol |
| Others vegetal and non-vegetal marker | Bovinocidin  Furanone, 5-(1-methylethyl)- |

| **NS SU 302 Aa** | |
| --- | --- |
| Sugars | Arabinose  Sedoheptulose |
| Amino acids | Alanine  Arginine  Threonine |
| Fatty acids | 11-Octadecenoic acid  9,12-Octadecadienoic acid  9-Octadecenoic acid  Docosanoic acid  Dodecanoic acid  Hexadecanoic acid  Octadecanoic acid  Tridecanoic acid |
| Alcohols | 1-Octanol  3-Nonen-1-ol |
| Terpens and derivatives | beta-copaene  Carveol  Cycloartanol  Linalool  Menthadiene  Squalene |
| Steroidal compounds | 3-Chloro-5-cholestene |

| **NS SU 302 Ab** | |
| --- | --- |
| Sugars | Galactose |
| Amino acids | Serine |
| Fatty acids | 11-Octadecenoic acid  2-Pentenoic acid  9,12-Octadecadienoic acid  9-Octadecenoic acid  Butanoic acid  Eicosapentaenoic acid  Decanoic acid  Docosanoic acid  Hexanoic acid  Octanoic acid  Triacontanoic acid |
| Alcohols | 1-Heptanol  Tetracosanol-1 |
| Terpens and derivatives | Citronellol |
| Phenolic compounds and derivatives | Cyclopentanocoumarine  Benzoic acid |
| Others vegetal and non-vegetal marker | Artemisia ketone  Succinic acid |

| **NS SU 304 Aa** | |  |
| --- | --- | --- |
| Amino acids | Alanine | |
| Fatty acids | 11,14-Eicosadienoic acid  9-Octadecenoic acid  13-Docosenoic acid | |
| Terpens and derivatives | 1,2-Dihydrolinalool | |
| Phenolic compounds and derivatives | Dihydrocoumarin | |
| Steroidal compounds | Cholest-5-en-3-ol | |

| **NS SU 307** | |
| --- | --- |
| Sugars | Fucose |
| Amino acids | Valine |
| Fatty acids | 13-Docosenoic acid  9,12-Octadecadienoic acid  9-Octadecenoic acid  Decanoic acid  Dodecanoic acid  Eicosapentaenoic acid  Heptacosanoic acid  Hexadecanoic acid  Triacontanoic acid |
| Alcohols | 1-Hexacosanol  1-Octanol  Tetracosanol |
| Terpens and derivatives | Citronellol  Ocimenolo |
| Others vegetal and non-vegetal marker | Furanone, dihydro-5-methyl- |

| **NS SU 309 Aa** | |
| --- | --- |
| Sugars | Galactosan  Galactose  Inositol  Rhamnose |
| Amino acids | Valine |
| Fatty acids | 13-Docosenoic acid  9,12-Octadecadienoic acid  9-Octadecenoic acid  Decanoic acid  Eicosapentaenoic acid  Pentadecanoic acid  Pentanoic acid  Tetradecanoic acid |
| Alcohols | 1-Heptanol  1-Octanol  2-Octenol |
| Terpens and derivatives | Menthene |
| Others vegetal and non-vegetal marker | Glucobrassicin |

| **NS SU 310 Aa** | |
| --- | --- |
| Sugars | Rhamnose |
| Fatty acids | 13-Docosenoic acid  9,12-Octadecadienoic acid  9-Octadecenoic acid  Butanoic acid  Eicosapentaenoic acid  Octanoic acid  Pentanoic acid  Tetradecanoic acid  Tridecanoic acid |
| Alcohols | 1-Octanol  3-Nonen-1-ol  Tetracosanol-1 |
| Terpens and derivatives | beta-Pinene  Citronellol |
| Phenolic compounds and derivatives | Catechol  Cyclopentanocoumarine |
| Others vegetal and non-vegetal marker | Glucobrassicin |

| **NS SU 311 Aa** | |
| --- | --- |
| Sugars | Sedoheptulose |
| Amino acids | Alanine  Serine |
| Fatty acids | 6-Octadecenoic acid  9,12-Octadecadienoic acid  9-Octadecenoic acid  Decanoic acid  Heptacosanoic acid  Octanoic acid  Pentadecanoic acid  Pentanoic acid  Tetradecanoic acid  Tridecanoic acid |
| Alcohols | 3-Nonen-2-ol |
| Vitamins | Folic Acid |
| Terpens and derivatives | Citronellol  Linalool  Squalene |

| **NS SU 316 Aa** | |
| --- | --- |
| Sugars | Galactose |
| Amino acids | Asparagine  Lysine  Valine |
| Fatty acids | 6-Octadecenoic acid  9,12-Octadecadienoic acid  9-Octadecenoic acid  Butanoic acid  Decanoic acid  Eicosapentaenoic acid  Hexadecanoic acid  Octadecanoic acid  Octanoic acid  Pentanoic acid  Triacontanoic acid  Tridecanoic acid |
| Alcohols | 1-Heptanol  4-Dodecanol  Tetracosanol |
| Terpens and derivatives | Artemisia alcohol  Citronellol  Limonene  Linalool |
| Phenolic compounds and derivatives | Cyclopentanocoumarine  Homogentisic acid |
| Others vegetal and non-vegetal marker | Furaneol  Succinic acid |

| **NS SU 318 Aa** | |
| --- | --- |
| Sugars | Inositol  Lactose |
| Amino acids | Asparagine  Serine |
| Fatty acids | 9,12-Octadecadienoic acid  9-Octadecenoic acid  Decanoic acid  Docosanoic acid  Eicosapentaenoic acid  Eicosatrienoic acid  Hexadecanoic acid  Octadecanoic acid  Pentanoic acid  Tetradecanoic acid  Tridecanoic acid |
| Alcohols | 1-Heptanol  Tetracosanol |
| Terpens and derivatives | Citronellol |
| Phenolic compounds and derivatives | Benzoic acid  Cyclopentanocoumarine  Homogentisic acid |
| Others vegetal and non-vegetal marker | Cholesterol |

| **NS SU 319** | |
| --- | --- |
| Sugars | Galactose  Inositol  Lactose |
| Amino acids | Alanine |
| Fatty acids | 13-Docosenoic acid  6-Octadecenoic acid  9,12-Octadecadienoic acid  9-Octadecenoic acid  Decanoic acid  Eicosapentaenoic acid  Heptacosanoic acid  Hexanoic acid  Octadecanoic acid  Tetradecanoic acid  Triacontanoic acid |
| Alcohols | 1-Octanol  2-Nonen-1-ol  Tetracosanol |
| Alcaloids and derivatives | Acetyl betonicine |
| Terpens and derivatives | Citronellal  Linalool |
| Phenolic compounds and derivatives | Cyclopentanocoumarine  Homogentisic acid |
| Others vegetal and non-vegetal marker | Cholesterol  Glucobrassicin |
| Combustion marker | Cyclopentanaphthalene |

| **NS SU 321** | |
| --- | --- |
| Sugars | Fucose  Galactopyranoside  Glucose  Inositol |
| Amino acids | Alanine  Arginine  Asparagine  Lysine  Serine  Threonine |
| Fatty acids | 11-Octadecenoic acid  13-Docosenoic acid  6-Octadecenoic acid  9,12-Octadecadienoic acid  9-Octadecenoic acid  Butanoic acid  Cyclopentaneundecanoic acid  Decanoic acid  Docosanoic acid  Eicosanoic acid  Eicosapentaenoic acid  Eicosatrienoic acid  Heptacosanoic acid  Hexadecanoic acid  Octadecanoic acid  Pentadecanoic acid  Pentanoic acid  Tetradecanoic acid  Tridecanoic acid |
| Alcohols | 1-Heptanol  1-Tetracosanol  3-Dodecanol  3-Nonen-1-ol  Cucumber alcohol |
| Alcaloids and derivatives | Stachydrine |
| Terpens and derivatives | alpha-Bergamotene  alpha-Farnesene  alpha-Ocimene  beta-Myrcene  beta-Terpinol  Aristolene  Bergamiol  Bornanone  Carvone  Camphene  Carene  Citronellal  Geraniol  Elemene  Eucalyptol  Isopinocampheol  Limonene  Linalool  Nerolidol  Squalene |
| Steroidal compounds | Cholesterol |
| Others vegetal and non-vegetal marker | Artemisia ketone  Octalactone  Glucobrassicin  Santolina triene  Sorbic acid  Succinic acid |

| **NS SU 322** | |
| --- | --- |
| Amino acids | Alanine  Lysine |
| Fatty acids | 11,14-Eicosadienoic acid  6-Octadecenoic acid  7-Hexadecenoic acid  9-Hexadecenoic acid  9-Octadecenoic acid  Butanoic acid  Cyclopentaneundecanoic acid  Decanoic acid  Dodecanoic acid  Eicosapentaenoic acid  Eicosatrienoic acid  Octadecanoic acid  Pentacosanoic acid  Pentanoic acid  Tetradecanoic acid  Tridecanoic acid |
| Alcohols | 1-Heptanol  1-Octanol  3-Nonen-1-ol |
| Terpens and derivatives | Dihydrocitronellol |
| Others vegetal and non-vegetal marker | Glucobrassicin |

| **NS SU 325** | |
| --- | --- |
| Sugars | Galactopyranose  Glucitol  Inositol  Sedoheptulose |
| Amino acids | Alanine  Serine |
| Fatty acids | 6-Octadecenoic acid  9,12-Octadecadienoic acid  9-Octadecenoic acid  Butanoic acid  Decanoic acid  Eicosatrienoic acid  Heptacosanoic acid  Hexadecanoic acid  Octadecanoic acid  Pentanoic acid  Tetradecanoic acid  Tridecanoic acid |
| Alcohols | Tetracosanol |
| Terpens and derivatives | Citronellol  Squalene |
| Others vegetal and non-vegetal marker | Glucobrassicin |

| **NS SU 326 Aa** | |
| --- | --- |
| Sugars | Galactosan  Galactopyranose  Glucofuranose |
| Amino acids | Alanine |
| Fatty acids | 7-Hexadecenoic acid  9,12-Octadecadienoic acid  9-Hexadecenoic acid  9-Octadecenoic acid  Butanoic acid  Decanoic acid  Docosahexanoic acid  Docosanoic acid  Eicosatrienoic acid  Heptacosanoic acid  Octadecanoic acid  Pentanoic acid  Tetradecanoic acid  Triacontanoic acid  Tridecanoic acid |
| Alcohols | 1-Heptanol  Tetracosanol |
| Terpens and derivatives | Dihydrocitronellol |
| Phenolic compounds and derivatives | Homogentisic acid |
| Others vegetal and non-vegetal marker | Acetovanillone  Glucobrassicin |

| **NS SU 327 Sa** | |
| --- | --- |
| Sugars | Sedoheptulose |
| Amino acids | Alanine  Citrulline  Lysine |
| Fatty acids | 6-Octadecenoic acid  7-Hexadecenoic acid  9,12-Octadecadienoic acid  9-Octadecenoic acid  Butanoic acid  Cyclopentaneundecanoic acid  Decanoic acid  Docosanoic acid  Dodecanoic acid  Eicosanoic acid  Eicosapentaenoic acid  Glutaric acid  Heptacosanoic acid  Hexadecanoic acid  Octadecanoic acid  Pentanoic acid  Tetradecanoic acid |
| Alcohols | 1-Heptanol  1-Octanol  Tetracosanol |
| Phenolic compounds and derivatives | Coumarin  Homogentisic acid |
| Steroidal compounds | Cholesterol |

| **NS SU 336 Aa** | |  |
| --- | --- | --- |
| Fatty acids | 9-Octadecenoic acid  Docosahexanoic acid  Hexadecanoic acid  Octadecanoic acid  Triacontanoic acid  Tridecanoic acid | |
| Terpens and derivatives | Linalool  Pinanol | |
| Phenolic compounds and derivatives | Dihydrocoumarin | |
| Steroidal compounds | Cholest-5-en-3-ol | |

| **NS SU 341 Aa** | |
| --- | --- |
| Sugars | Dulcitol  Inositol |
| Amino acids | Alanine  Lysine  Serine |
| Fatty acids | 2-Butenoic acid  6-Octadecenoic acid  9,12-Octadecadienoic acid  9-Octadecenoic acid  Butanoic acid  Decanoic acid  Docosahexanoic acid  Docosanoic acid  Heptacosanoic acid  Hexadecanoic acid  Octadecanoic acid  Tetradecanoic acid  Tridecanoic acid |
| Alcohols | 1-Heptanol  1-Hexacosanol  3-Nonen-1-ol |
| Terpens and derivatives | Bergamiol  Linalool |
| Phenolic compounds and derivatives | Cyclopentanocoumarine  Homogentisic acid |
| Others vegetal and non-vegetal marker | Artemiseole  Glucobrassicin  Malonic acid  Octalactone |
| Combustion marker | Naphthalene |

| **NS SU 344 Aa** | |
| --- | --- |
| Sugars | Inositol  Sedoheptulose |
| Amino acids | Alanine  Creatine  Lysine |
| Fatty acids | 10-Undecenoic acid  11,14-Eicosadienoic acid  11-Octadecenoic acid  6-Octadecenoic acid  9-Hexadecenoic acid  9-Octadecenoic acid  Butanoic acid  Decanoic acid  Docosahexanoic acid  Docosanoic acid  Eicosanoic acid  Glutaric acid  Heneicosanoic acid  Heptacosanoic acid  Hexanoic acid  Octadecanoic acid  Pentadecanoic acid  Pentanoic acid  Tetracosanoic acid  Tetradecanoic acid  Triacontanoic acid  Tridecanoic acid |
| Alcohols | 1-Dodecanol  2-Undecenol  3-Heptanol |
| Alcaloids and derivatives | 4-Piperidinone  Stachydrine |
| Terpens and derivatives | Artemisia alcohol  Citronellal  Linalool  Selinene  Thymol |
| Phenolic compounds and derivatives | Benzoic acid  Gentisic acid  Homogentisic acid  Hydrocinnamic acid |
| Steroidal compounds | Cholesterol |
| Others vegetal and non-vegetal marker | 2,6-Lupetidine  Furanone, 5-butyldihydro-4-methyl- |

| **NS SU 346 Ab** | |
| --- | --- |
| Sugars | Galactosan |
| Amino acids | Serine |
| Fatty acids | 11,14-Eicosadienoic acid  6-Octadecenoic acid  9-Octadecenoic acid  13-Docosenoic acid  Decanoic acid  Docosanoic acid  Heptacosanoic acid  Hexadecanoic acid  Octadecanoic acid  Pentanoic acid |
| Alcaloids and derivatives | Ephedrine |
| Terpens and derivatives | Squalene |
| Others vegetal and non-vegetal marker | Glucobrassicin |

| **NS SU 347 Ab** | |
| --- | --- |
| Sugars | Galactose  Galactosan  Inositol |
| Amino acids | Alanine  Asparagine  Lysine  Serine |
| Fatty acids | 9,12-Octadecadienoic acid  9-Octadecenoic acid  Butanoic acid  Cyclopentaneundecanoic acid  Decanoic acid  Eicosanoic acid  Heptacosanoic acid  Hexadecanoic acid  Pentanoic acid  Tridecanoic acid |
| Alcohols | 1-Heptanol  1-Octanol |
| Vitamins | Aspartic acid |
| Terpens and derivatives | alpha-Bisabolene  Citronellol  Linalool |
| Combustion marker | Naphthalene |

| **NS SU 356** | |
| --- | --- |
| Sugars | Galactopyranose  Galactose |
| Amino acids | Alanine  Asparagine  Serine |
| Fatty acids | 9,12-Octadecadienoic acid  9-Octadecenoic acid  Cyclopentaneundecanoic acid  Docosanoic acid  Dodecanoic acid  Heptacosanoic acid  Octadecanoic acid  Pentanoic acid |
| Alcohols | 1-Heptanol |
| Alcaloids and derivatives | Ephedrine  Muscimol |
| Terpens and derivatives | Linalool |
| Phenolic compounds and derivatives | Homogentisic acid  Pyrogallol |
| Others vegetal and non-vegetal marker | Glucobrassicin  Succinic acid |
