# Supplementary material for: A multidisciplinary approach for investigating dietary and medicinal habits of the Medieval population of Santa Severa (7th-15th centuries, Rome, Italy)
Source: PLoS One. 2020 Jan 28;15(1):e0227433. doi: 10.1371/journal.pone.0227433 (PMC6986732; doi:10.1371/journal.pone.0227433)
Supplement: S6 Table — For each sample. chronology, sample size and relative reference were reported [7, 8, 19, 20, 21, 86, 90, 91, 144–145]. (DOCX) [file pone.0227433.s006.docx]

**S6 Table.** Mean of δ^13^C and δ^15^N of human samples recovered in Santa Severa archaeological site and in other coeval Italian sites from literature. For each sample the chronology, the sample size as well as the reference are reported.

| **Archaeological site** | **Chronology (centuries)** | **Sample size (N)** | **mean δ^13^C** | **SD** | **mean δ^15^N** | **SD** | **Reference** |
| --- | --- | --- | --- | --- | --- | --- | --- |
| Santa Severa | 7th-15th | 112 | -19.0 | 0.5 | 9.3 | 1.1 | present research |
| Trino Vercellese | 8th-13th | 28 | -19.1 | 0.7 | 9.2 | 0.8 | [7] |
| Pava Pieve, Siena | 8th | 19 | -19.3 | 0.9 | 9.2 | 0.6 | [144] |
| Piazza Madonna di Loreto, Rome | 8th | 13 | -20.1 | 0.8 | 8.6 | 0.8 | [145] |
| Romans d'Isonzo | 6th-7th | 42 | -16.4 | 0.7 | 8.4 | 0.5 | [90] |
| Cividale Gallo | 6th-7th | 7 | -16.5 | 1.3 | 8.7 | 1.5 | [90] |
| Cividale S. Stefano | 6th-7th | 11 | -17.3 | 1.1 | 8.5 | 1.0 | [90] |
| Mainizza | 10th-11th | 16 | -15.9 | 1.4 | 7.7 | 1.1 | [90] |
| Cosa | 11th-13th | 26 | -17.7 | 0.6 | 8.8 | 1.1 | [86] |
| Albano Laziale, Rome | 11th-13th | 24 | -18.7 | 0.5 | 8.4 | 1.1 | [8] |
| Montella | 13th-15th | 48 | -19.7 | 0.7 | 7.1 | 1.1 | [91] |
| Colonna, Rome | 8th-10th | 58 | -18.9 | 0.4 | 7.7 | 0.6 | [19] |
| Allumiere, Rome | 15th-17th | 41 | -19.1 | 1.0 | 8.5 | 1.3 | [20] |
| Leopoli-Cencelle, Viterbo | 12th-15th | 76 | -19.2 | 0.4 | 8.9 | 1.0 | [21] |
